# Flexible Working Arrangements and Fertility Intentions: A Survey Experiment in Singapore

**DOI:** 10.1007/s10680-024-09719-1

**Published:** 2024-11-21

**Authors:** Senhu Wang, Hao Dong

**Affiliations:** 1 National University of Singapore, Singapore, Singapore; 2 Peking University, Beijing, China

**Keywords:** Flexible working arrangements, Fertility intentions, Work–family conflicts, Occupation, Survey experiment

## Abstract

This study examines how young, unmarried, working people’s fertility intention is shaped by future scenarios where flexible working arrangements (FWAs) are the default. The unmarried population remains to be an under-studied part of the working population at reproductive ages, who nevertheless becomes increasingly significant for fertility research due to rising ages at marriage and the first birth. Despite significant public anticipation regarding the potential of FWAs to facilitate work–family balance and fertility, there is little research on the effects of FWAs on fertility intentions. We conduct a population-based vignette survey experiment to identify the causal effects of FWAs by randomly manipulating three scenarios of FWAs policy changes—reducing hours, increasing work-schedule flexibility, and increasing workplace flexibility—in Singapore, where both overwork norm and low fertility co-exist. All three types of FWAs improve fertility intentions. The effects are especially substantial for women, for which anticipated work–family conflict is an important mediator. Moreover, FWAs matter particularly to those in professional and managerial occupations. These findings call for policies facilitating a more family-friendly environment to tackle low fertility in the future of work.

**Supplementary Information:**

The online version contains supplementary material available at 10.1007/s10680-024-09719-1.

## Introduction

The fertility of many developed countries has been low and delayed further over the past several decades (Lee et al., 2014; McDonald, 2006). Among the developed countries, Singapore has experienced one of the most significant fertility declines in the world (Yeung et al., 2018). Singapore’s total fertility rate (TFR) has declined from 5.8 to 1.1 from 1960 to 2020, while the TFR remains at 1.5 in Western Europe and 1.6 in North America (The World Bank, 2020). One of the key reasons for declining fertility is the postponement of childbearing, driven by the rising age at first marriage and parenthood. Singapore is, however, not alone in Asia since many East Asian countries and regions have experienced dramatic fertility declines toward very low fertility levels (Raymo et al., 2015). It has been widely argued that very low fertility may result in various social problems, featuring population aging and labor force shortage (Lee et al., 2014; Raymo et al., 2015; Yeung et al., 2018).

Meanwhile, recent years have witnessed the rise of flexible working arrangements (FWAs). FWAs refer to work arrangement options that entail employees’ control over how much, when, or where they work, such as reduced hours, flexible schedules, and teleworking (Chandola et al., 2019; Chung & van der Lippe, 2020). Especially during the COVID-19 pandemic, FWAs have been temporarily expanded as a solution to allow organizations to continue operations and preserve jobs all over the world. There has been an increasing public clamor to normalize and legitimize the use (rather than the request) of FWAs in the future because they are believed to help address work–family conflicts and other challenging social problems such as low fertility rates.

However, research on the influence of FWAs on family behavior remains limited. It is difficult to disentangle the roles of workplace policies and marriage/fertility trends due to endogeneity problems. As social policies generally reflect dominant public views, a country’s work–family policies tend to mirror and reinforce its residents’ existing family values and attitudes. Similarly, individuals with low fertility intentions tend to prioritize their career development by working long hours and accommodating their employers' work schedules and place requirements.

Adding to the emerging literature of experimental studies on fertility intentions (Lappegård et al., 2022; Vignoli et al., 2022), this research uses a population-based vignette survey experiment to examine how changing the existing (over)work norms through government-initiated policies such as FWAs affects fertility intention. Specifically, this study randomly assigned the respondents into three treatment groups and one control group. Respondents in the treatment groups were presented hypothetical government policy changes toward three common FWAs options: reduced hours, flexible schedule, and flexible place (Chandola et al., 2019; Li & Wang, 2022; Wang & Cheng, 2023), while those in the control group were shown the information of existing policies regarding such work arrangements. The survey experiment was conducted in Singapore, an ideal research site with common overwork norms and low fertility rates.

In doing this experiment, we first identify how people’s fertility intention varies in response to changing work flexibility by experimentally manipulating the three types of FWAs. In this study, we focused on the 5-year fertility intention, a practical measure that captures young people's near-term fertility plans, which largely align with realistic life transitions particularly in contexts like Singapore where childbearing is often delayed. Then, we explicitly examine the extent to which the effects of the FWAs on fertility intention are mediated by changes in the respondent’s anticipated work–family conflict. Moreover, we further explore the moderating role of occupational class, expecting to find that the effects of FWAs on fertility intention are particularly pronounced in professional and managerial (hereafter “professional”) occupations because of their different job demands, work norms, and work–family conflict levels from non-professional occupations.

This study contributes to the previous literature in the following ways. First, this research is the first study to implement a randomized information treatment approach to simulate future work scenarios where FWAs are commonplace. This innovative methodological approach enables us to bridge and illuminate the causal relationship between FWAs and fertility intentions, effectively connecting two previously distinct lines of research. By theorizing and empirically assessing how FWAs could potentially address the low fertility dilemma, our research offers a fresh lens through which to comprehend the dynamics at play. Additionally, our analysis delves into the varied impacts of FWAs across different occupational sectors, shedding light on the mechanisms through which work policies influence fertility decision-making processes. This nuanced exploration contributes significantly to our understanding of how individuals from diverse socioeconomic backgrounds navigate the intersection of work flexibility and family planning. Through this method, we not only test a probable solution to the low fertility challenge but also enrich the dialogue between work policy design and fertility intentions, highlighting the importance of considering heterogeneous effects in policy formulation.

Second, more importantly, our study extends our knowledge to an increasingly significant yet under-studied part of the population at reproductive ages in Asia, that is, the unmarried population (Raymo et al., 2015; Yeung et al., 2018). While previous studies mainly examined the fertility intention among the married since their definition of the at-risk population often hinges on marriage, (Raymo et al., 2015; Yeung et al., 2018), the unmarried population has increased steadily and rapidly in many Asian countries, including Singapore (for example, based on the official statistics, 56.8% of those aged 20–39 remain unmarried in Singapore in 2022). This focus on the unmarried population is especially pertinent given the traditional norm in many Asian societies where marriage precedes childbearing. Thus, we are facing a common bimodal pattern regarding marriage and fertility in many Asian populations, including Singaporeans: On the one hand, most childless adults are also unmarried; on the other hand, the majority of married couples tend to have at least two children. By focusing on the unmarried, our study becomes particularly valuable in filling a potentially significant knowledge gap with new evidence about the unmarried, a silent minority—or even majority—of the population at reproductive ages. The findings enable us to develop a more comprehensive understanding of the shaping of their fertility intentions and promising pro-natal policy intervention tools.

## Background

### Fertility intentions in very-low-fertility Asian contexts

As a concrete predictor of fertility outcomes, fertility intention has been widely surveyed and studied to understand recent fertility decline trends (Duvander et al., 2020; Wang & Gong, 2023). Systematic evidence demonstrates a substantial association between fertility intention and behavior (Harknett & Hartnett, 2014). When asked about the short-term, fertility intentions are strongly associated with realized fertility (e.g., Liefbroer, 2005; Harknett & Harknett, 2014; Miller & Pasta, 1995; Philipov, 2009). Moreover, studying the structural obstacles facing people in shaping and realizing their fertility intentions can provide essential insights into our understanding of low fertility. According to Brinton et al. (2018), in very-low-fertility European countries such as Austria, Germany, Hungary, and Italy, individuals tend to relate their fertility intentions even more closely to conditions constraining the realization of such intentions than their counterparts in moderately low-fertility countries. This comparison provides important insights to pay attention to relevant practical constraints when studying fertility intentions in other very-low-fertility countries, like the present study of FWAs in Singapore.

However, Singapore—and probably other very-low-fertility Asian countries, too—has a striking difference in fertility behavior from very-low-fertility European countries. Singapore and East Asian countries tend to have rare non-marital births (Raymo et al., 2015; Yeung et al., 2018). In these Asian countries, marriage is culturally deemed as a pre-condition for fertility, and the proportion of childless among the married population is considerably lower than in other countries where non-marital births are more prevalent.

This Asian pattern of the close link between marriage and fertility merits our attention because it has two implications for understanding fertility intentions in very-low-fertility Asian contexts. First, while studies on Western countries often regard marriage intentions and fertility intentions as two distinct subjects, it is reasonable to suppose a much stronger association between the two intentions in very-low-fertility Asian countries, given their rare non-marital births in the present (Raymo et al., 2015; Yeung et al., 2018). Arguably, there could be a non-trivial proportion in those Asian populations who intended to marry because of the intention to have children—and vice versa.

Second, because of the close inter-relationship between marriage and fertility intentions in these very-low-fertility Asian countries, we should not just pay attention to the fertility intentions of married couples; more attention should be extended to unmarried Asian individuals. Brinton and Oh (2019) pioneered to capitalize on the narratives of unmarried individuals in Japan and Korea for a systematic understanding of the labor market, workplace norms, and gendered division of household labor on fertility decision-making. That being said, prior research on fertility intention commonly focuses on married or partnered individuals. One reason is probably the long convention underscored by classical studies on Western populations, such that fertility intentions predict real-life fertility more accurately among married or partnered individuals (e.g., Schoen et al., 1999; Westoff & Ryder, 1977).

For the unmarried in these Asian countries, if constraints for realizing their fertility intentions can be lifted, or the intentions themselves can be encouraged by specific pro-natal policies and institutions, the transition from singlehood to parenthood may become *relatively swift* because marriage is culturally bundled with childbearing and, therefore, less a constraint for them than their Western counterparts. This conjuncture highlights the distinctive premise for studying fertility intentions of the unmarried in these very-low-fertility Asian countries that differ qualitatively from many other countries, where marriage and reproduction involve separate sets of considerations for decision-making.

Moreover, the significance of unmarried people’s fertility intentions becomes even more significant in the presence of their rapidly growing proportion among the young population in Asian countries, thanks to rising ages at first marriage and the retreat of marriage among young generations (Raymo et al., 2015; Yeung et al., 2018). In Singapore, the unmarried have already accounted for more than half of the age 20–39 population in 2022. Thus, the focus on the unmarried population in this study is especially pertinent given that not entering into marriage is a significant barrier to fertility in many Asian societies, whereas most married couples tend to have at least two children (Yeung & Hu, 2020). By focusing on the unmarried, a rising majority of the actively reproductive population in very-low-fertility Asia, our study makes important contributions to facilitate a more comprehensive understanding of fertility intentions and promising pro-natal policy intervention tools.

### Theories of fertility costs

In light of the very low fertility rates and intentions in Asian contexts, economic theories suggest that decisions about fertility involve weighing the utility against the costs (Becker, 1960; Leibenstein, 1975). When the perceived costs outweigh the anticipated benefits, individuals are more likely to postpone or forego having children. These costs are categorized into two types. While the direct economic costs include healthcare, education, and child-rearing expenses, the indirect opportunity costs refer to the potential benefits an individual foregoes when choosing one option over alternatives (Billingsley & Ferrarini, 2014; Harknett et al., 2014). In the context of fertility decisions, the opportunity costs for working individuals often involve the loss of income, career advancement, or personal development that comes with parenthood.

In contemporary societies, the opportunity costs of childbearing and rearing are particularly significant and have become increasingly relevant. Such opportunity costs matter particularly for working women, who remain to bear a disproportionate share of childcare and domestic responsibilities and, therefore, often have to experience disadvantages in labor earnings and career development (Billingsley & Ferrarini, 2014; Brinton et al., 2018; Kong & Dong, 2024). The rising importance of indirect opportunity costs in fertility decisions, especially for women, can be attributed to several societal changes. In recent decades, women’s participation in higher education and the workforce has surged, assortative marriages by a couple’s socioeconomic status have increased, and societal norms and expectations regarding gender roles and family life have evolved, with increased emphasis on dual-earner households (Chung & van der Horst, 2018; Dong & Xie, 2023; Wang & Li, 2022).

However, despite a significant improvement in gender equity within individual-oriented institutions such as education and employment, there remains persistent gender inequity within family-oriented institutions, where traditional gender roles in the workplace and home prevail (McDonald, 2000, 2016). This disparity results in women shouldering a disproportionate burden of domestic tasks, even as they engage in paid employment. This dual burden of work and family roles exacerbates work–family conflict, particularly as women’s opportunity costs in the labor market increase with their higher educational and career aspirations (Brinton & Oh, 2019; Nagase & Brinton, 2017). This gap in gender equity between individual and family spheres suggests that women are caught in a challenging position, striving to balance their professional ambitions with family responsibilities. Thus, as women attain higher levels of education and more significant career opportunities, the potential sacrifices in terms of career progression and earnings due to childbearing become more substantial (Brinton & Oh, 2019; Gong & Wang, 2022). The heightened opportunity costs, especially for women, are thought to be a crucial reason for low fertility rates and intentions.

### Flexible work arrangements, work–family conflict, and fertility intention

In recent years, the rise of flexible work arrangements (FWAs) provides an opportunity for individuals to better negotiate their work and family lives. Especially during the Covid-19 pandemic, various types of FWAs have been introduced to allow employees to work remotely (flexible workplace), vary their starting and ending times (flexible work hours), offset overtime hours with short periods of leave, work a compressed week or annualized hours, work part-time, or job share (flexible workload). In the post-Covid period, there has been an increasing public advocacy to normalize the use (rather than just the request) of FWAs to address some of the most challenging social issues, including low fertility (CIPD, 2023).

Work–family conflict emerges as a pivotal mediator in the nexus between FWAs and fertility intentions, underpinned by theories and empirical research (Chung & van der Lippe, 2020; Kelly et al., 2014; Moen et al., 2016). This theoretical framework of work–family conflict posits that individuals navigating concurrent responsibilities across work and family spheres often encounter conflicting role pressures due to the disparate rules, thought processes, and behaviors demanded by each domain (Greenhaus & Beutell, 1985).

Specifically, the conflict manifests in three distinct forms (Greenhaus & Beutell, 1985). First, time-based conflict occurs when the time dedicated to work interferes with family responsibilities, or vice versa, such as extended working hours clashing with family care duties (Kamerāde et al., 2019; Wang & Cheng, 2023; Kong & Dong, 2024). FWAs offer a potential solution by allowing individuals to tailor their work schedules to better accommodate family needs, thereby reducing time-based conflict. Second, strain-based conflict refers to stress or anxiety from one domain can spill over and adversely affect the other, diminishing an individual’s capacity to fulfill their roles effectively (Li & Wang, 2022). FWAs, by providing a mechanism to manage work stressors more flexibly, might lessen the strain experienced in the family domain, aiding in the reconciliation of work and family life. Third, behavior-based conflict occurs when the behaviors required in one domain make it challenging to meet the demands of the other. FWAs can facilitate a more seamless transition between these roles by offering the autonomy to navigate the demands of both domains more harmoniously (Chung & van der Lippe, 2020; Chung et al., 2021). By addressing the underlying work–family conflicts that discourage individuals from starting or expanding their families, FWAs directly contribute to creating an environment where the intention to have children becomes more feasible and desirable.

We also expect that the potentially positive effect of FWAs on fertility intention may be stronger for women. Increasing evidence suggests that the prevalence of the overwork norm, as characterized by long and inflexible working hours, is an important institutional constraint for marriage or fertility intention (Begall & Mills, 2011; Kim & Lee, 2019; Yu & Hara, 2020). For example, Begall and Mills find that women with higher levels of work control are more likely to intend to have children, whereas higher levels of job strain tend to lower their fertility intention, especially in countries with low childcare availability (Begall & Mills, 2011). In addition, Yu and Hara’s research shows that job autonomy can significantly improve marriage intention, especially for men (Yu & Hara, 2020). Similarly, Kim and Lee show that working time reduction policies could lower male workers’ risk of divorce (Kim & Lee, 2019).

Moreover, the pro-natal effects of the FWAs may vary by occupation. First, it is argued that overwork norm is more prevalent in professional occupations, leading to higher levels of work–family conflicts and mental stress (Schieman et al., 2006, 2009a, 2009b). On the one hand, this is because in professional occupations, workers’ productivity is hard to measure in standardized ways, so working long hours without interruptions becomes a more salient sign of their work commitment (Cha, 2010; Chung, 2020). On the other hand, the labor market restructuring over the last decades has resulted in the polarization of working hours, further exacerbating the overwork norm in high-class occupations (Wang et al., 2022). Second, the culture of “intensive parenting” is more salient in professional occupations, leading to higher material and opportunity costs of fertility (Ishizuka, 2019). For example, Lareau (2011) finds that the parenting style in middle-class families involves ‘‘concerted cultivation’’, characterized by intense parental engagement in children’s education, development, and leisure activities (Lareau, 2011).

### The Settings of Singapore

In Singapore, marriage and fertility are regarded as the foundation of family and society. Over the last several decades, Singapore’s demographic landscape has experienced significant transformations, echoing shifts reminiscent of those in European countries during the late twentieth century. The nation’s TFR has declined to an alarming low of 1.1 in 2020, signaling a critical juncture in its demographic evolution.

Given the close link between marriage and fertility in Singapore and the very low non-marital birth rate (around 2%) in Singapore, it is argued that the low fertility rates are accompanied by rising first-marriage ages and an increasing proportion of singles. For example, the last decade has witnessed a steady increase in the median age at first marriage (from 30.1 to 30.5 for men and 28.0 to 29.1 for women), according to the Singapore Census in 2021 (Singapore Department of Statistics, 2020). The proportion of singles has also increased across all age groups over the past decade, especially among those aged 25 to 34. For example, the proportion of singles aged 25 to 29 went up from 74.6% to 81.6% for men and from 54 to 69% for women. Also, among those aged 30 to 34, the proportion increased from 37.1% to 41.9% for men and 25.1% to 32.8% for women.

In response, various family policies in Singapore are designed to support marriage, childbearing, and work-life balance, often with an emphasis on encouraging higher fertility rates (Ministry of Social & Family Development, 2024). Key initiatives include financial incentives such as the Baby Bonus Scheme, which provides cash gifts and savings contributions for parents, and the Child Development Account (CDA), which helps parents save for their children's education and healthcare needs. Singapore also offers paid parental leave—16 weeks for mothers and 2 weeks for fathers—and subsidies for childcare and preschool education through the Anchor Operator Scheme. Housing policies also prioritize families, offering subsidized housing grants for newlywed couples. Despite these policies, fertility rates remain low due to social factors such as career prioritization, cost of living, and delayed marriages.

This decline can be attributed to a multifaceted array of factors, each interplaying to shape the current fertility patterns observed within the city-state. The characteristics of the youth labor market play a significant role in this demographic shift. The transition from education to employment is a critical phase for young adults, and the nature of this transition can significantly impact decisions around family planning (Yeung & Hu, 2018; Yeung & Mu, 2019). In Singapore’s highly competitive job market, where job security and career progression are paramount concerns, many Singaporeans, particularly women, are choosing to postpone starting a family (Yeung & Yap, 2013). This delay is not merely a demographic statistic but is deeply rooted in the socioeconomic fabric of Singaporean society. Women’s increased participation in the labor market, a shift in traditional gender roles, and greater access to higher education have all contributed to this trend (Yeung et al., 2018). The pursuit of career advancement and personal development has, for many, taken precedence over early family formation.

Notably, Singapore is one of the most overworked countries in the world and has the longest working hours per week at around 45, with 23 percent of people working more than 48 h per week. Singapore's average working hours are much higher than in European countries, at around 35 h per week. Also, the maximum allowed working hours in Singapore can be up to 58 h, much higher than in Europe at 48 h. It has been reported that the overworking culture has undermined Singaporeans’ mental health and wellbeing and increased their feelings of burnout. The prevalence of overwork in Singapore reflects its “kiasu” (afraid to lose) culture and strong work ethic (Yeung & Hu, 2018; Yeung & Yap, 2013), which require employees to work long hours and devote their time and energy entirely to the jobs without any disruptions from non-work demands. In light of this, scholars call for reduced working hours and a more family-friendly working environment, suggesting that this may help solve some of the most pressing social issues, such as low marriage and fertility rates (Yeung & Hu, 2018).

## Research Questions and Hypotheses

Using a survey experiment design, this study aims to address the following three research questions and formulate three hypotheses based on the above theoretical discussion.

First, is a policy change advocating for FWAs as the default option in workplaces related to the fertility intentions of young unmarried men and women in Singapore? This research question explores how a policy change of FWAs to challenge the prevalent culture of overwork may shape young unmarried people’s fertility intentions. We pay attention to three dimensions of FWAs, including flexibility in workload, flexibility in work schedules, and flexibility in workplace, which have been documented in previous research. As there are no clear theoretical expectations about the differentiated impact of various FWAs on fertility intentions, we formulate an overall hypothesis without distinguishing types of FWAs. In addition, given the widely reported gender inequalities in the labor market and domestic domains, we also expect gender differences in the impact of FWAs on fertility intentions.

Hypothesis 1: A policy change advocating FWAs as the default option in workplaces increases fertility intentions for young unmarried men and women.

Hypothesis 2: The effects of FWAs on fertility intentions are more substantial for women than for men.

Second, to what extent can anticipated work–family conflict mediate the relationship between FWAs and fertility intentions? Given that work–family conflict is an important intervening mechanism underlying the relationship between FWAs and fertility intentions, the second research question aims to examine the role of work–family conflict in mediating the effect of FWAs on fertility intentions. Specifically, we expect that the policy change of FWAs increases fertility intentions by reducing anticipated work–family conflict and formulate the following hypothesis.

Hypothesis 3: Lower anticipated work-family conflict can mediate the effects of FWAs on fertility intentions.

Third, to what extent does the impact of FWAs vary with occupations? Given the widely documented occupational variations in job demands and workplace norms, the third research question explores how the impact of FWAs on fertility intentions varies across occupations. While there could be numerous heterogeneities in the FWAs’ impact to explore, we are particularly interested in such heterogeneity between professional occupations and non-professional occupations. This research interest is not just due to the conceptually natural connection between occupations and work policies like FWAs, but also motivated by the conventional differences in socioeconomic status, family behavior, and parenting practices between the two occupational classes. Specifically, those in professional and managerial occupations tend to invest more in child development and adopt intensive parenting styles (Gu, 2021). Specifically, we expect that the influence of FWAs on fertility intentions is stronger for those in professional occupations where there exist a strong overwork culture and intensive parenting styles, and thus formulate the following hypothesis.

Hypothesis 4: The effects of FWAs on fertility intentions are stronger in professional occupations than non-professional occupations.

## Methodology

### Experimental design

To examine the impacts of flexible working arrangements on fertility intentions, this study used a survey-experimental research design, which involves a randomized information treatment. As demonstrated by recent studies on fertility intentions, experiments have advantages in identifying the causal effects and mechanisms of theoretically sound factors on fertility intentions. The experimental designs work best for questions regarding future scenarios and resulting intentions, which have been particularly challenging to measure and analyze with observational data. The recent accumulation of experimental evidence already suggests such subjective consequences of perceptions about family policies and economic prospects have important implications for understanding the current situations and future changes in fertility. With a few exceptions (e.g., Gong & Wang, 2022), most experimental evidence has been drawn from western settings, of which the gap in the literature also motivates our current experimental study from a comparative perspective.

In our randomized information treatment, we have three treatment conditions and one control condition. The three treatment conditions are scenarios of near-future policy changes from existing policies that emulate three aspects of FWA, including flexibility in workload, flexibility in work schedules, and flexibility in workplaces, which are widely documented in previous literature and government policies (Chandola et al., 2019; Li & Wang, 2022). In such a family-friendly working environment, people in Singapore could work shorter hours (e.g., the average working hours per week in Europe), work with a more flexible schedule, or choose a workplace more flexibly (like in recent workplace trials) (Bloom et al., 2015; Fernandez-Lozano et al., 2020; Kamerāde et al., 2019). The control condition refers to the scenario of no near-future change from existing policies.

Conducting a national online survey with procedures detailed in the next section, we used a between-subjects experimental design to randomly allocate the respondents into these four conditions (vignettes): “control,” “reduced hours,” “flexible work schedule,” and “flexible workplace.” To ensure the information equivalence, each group was presented with a similar table showing the existing or future workplace policies and then asked about their planned family behavior. The control group was shown a table summarizing the existing policies regarding standard working hours (44 h per week), flexible work schedule, and flexible workplace (no legal regulations). The three treatment groups were presented with the policies of reduced working hours (from 44 to 36 h per week), flexible work schedule (at least two days per week), and flexible workplace (at least two days per week), respectively, with other workplace arrangements being unchanged. More details of the treatment can be found in Online Appendix Figures A1-A4.

### Survey and sample

We delegated a leading international survey company (i.e., Kantar) to conduct an online survey in Singapore in April 2022. This company maintains the largest national online panel (around 700,000 respondents) in Singapore, whose total population is 5.64 million in 2022. It employs various recruitment methods such as e-newsletter campaigns, opt-in email, co-registration, social media, as well as both internal and external affiliate networks to ensure the coverage of diverse population.

We draw a stratified random sample from the survey company’s online panel to select unmarried, childless and employed individuals aged 25–39 years in Singapore. The sampled respondents are invited to complete an online survey regarding their planned family behavior as well as social and demographic characteristics. The survey company incentivized participants with a points-based reward system, allowing respondents to convert their accumulated points into vouchers for online shopping. To ensure the statistical power of our sample, the recruitment procedure follows a quota sampling protocol, with which we pre-determined the quota for each sampling stratum according to the population distribution by gender and 5-year age group. To ensure experimental realism (Mutz, 2011), we conducted a manipulation check at the end of the questionnaire. Respondents were asked to choose the treatment information they had received from the four policy scenarios. Respondents who failed the manipulation check (around 30%) have been excluded from the analysis. The rate of passing manipulation checks is generally comparable with previous similar research (Pedulla & Thébaud, 2015). In addition, a series of quality checks (e.g., duplication of IP addresses, survey speedsters, honesty detector) were conducted to ensure the data quality.

The final analytic sample contains 1,092 respondents in total, with 274 in the control group, 271 in the “reduced hours” treatment group, 272 in the “flexible work schedule” treatment group, and 275 in the “flexible workplace” group. A probability weight was constructed and applied to all analyses to represent the young unmarried population in Singapore. Further analyses also show that the distributions of key variables in the sample, such as gender, age groups, and education levels, are similar to the distributions in Singapore Census data in 2020 (see Table A1). This confirms that our sample can be thought to generally represent the young unmarried people in Singapore.

**Table 1:** Weighted descriptive statistics and balance check

	Control group	Reduced hours	Flexible schedule	Flexible workplace	*F*/χ^2^ tests
Fertility intention, %					*p* = 0.006
Definitely no	12.09	5.21	6.19	6.97	
Probably no	21.68	11.87	15.35	16.50	
Not sure	23.93	31.38	27.47	23.90	
Probably yes	34.28	38.10	41.77	40.33	
Definitely yes	8.01	13.45	9.22	12.30	
Fertility intention, M	3.04	3.42	3.32	3.34	*p* < 0.001
Fertility intention, SD	(0.07)	(0.06)	(0.06)	(0.07)	
Work–family conflicts, M	2.95	2.64	2.75	2.72	*p* < 0.001
Work–family conflicts, SD	(0.05)	(0.05)	(0.04)	(0.05)	
Age groups, %					*p* = 0.955
25–29	54.94	53.98	54.48	51.53	
30–34	28.83	30.90	28.30	30.04	
35–39	16.22	15.13	17.23	18.42	
Gender, %					*p* = 0.918
Men	50.39	53.29	52.79	51.84	
Women	49.61	46.71	47.21	48.16	
Partnership, %					*p* = 0.954
Yes	47.29	48.49	46.76	48.98	
No	52.71	51.51	53.24	51.02	
Citizenship, %					*p* = 0.301
Citizens	87.78	83.46	87.02	83.05	
Permanent residents	12.22	16.54	12.98	16.95	
Education levels, %					*p* = 0.834
Below bachelor's degree	38.27	40.60	41.62	41.96	
Bachelor's degree	61.73	59.40	58.38	58.04	
Occupational class, %					*p* = 0.630
Non-professional	64.93	63.79	60.46	65.54	
Professional	35.07	36.21	39.54	34.46	
Normal working hours, M	41.09	40.72	41.04	41.83	*p* = 0.745
Normal working hours, SD	(0.57)	(0.58)	(0.57)	(0.47)	
Overtime, M	5.93	5.91	6.08	6.48	*p* = 0.731
Overtime, SD	(0.33)	(0.29)	(0.32)	(0.32)	
% work allowing flexible schedule, M	64.04	64.30	63.92	63.85	*p* = 0.973
% work allowing flexible schedule, SD	(29.51)	(28.82)	(29.04)	(29.40)	
% work allowing teleworking, M	52.06	49.87	50.73	50.62	*p* = 0.770
% work allowing teleworking, SD	(32.54)	(32.81)	(33.05)	(33.13)	
Number of observations	274	271	272	275	

Notes: % = Column proportions, M = Means, SD = Standard deviations. Column proportions may not add up to 100 due to rounding. Two-tailed *F* and *χ*^*2*^ tests were conducted for inter-group comparisons. Weights are constructed for national representativeness, according to the relevant proportions of each gender-age-education strata in the population

### Measures

The outcome variable is fertility intention, measured by the question: “Do you plan to have a child within the next five years?” Following the previous research (Billingsley & Ferrarini, 2014; Thomson & Brandreth, 1995), the question is treated and measured by a five-point interval scale where people’s fertility intention moves alongside a continuum ranging from “definitely no” to “definitely yes” (i.e., definitely no, probably no, not sure, probably yes, and definitely yes). The key explanatory variable is the treatment condition, distinguishing the aforementioned four categories (“control,” “reduced hours,” “flexible work schedule,” and “flexible workplace”).

As a theoretically important mediator of interest to our study, anticipated work–family conflicts are measured by four questions from a validated scale developed by Frone et al. (Frone et al., 1992). Specifically, respondents were asked to imagine themselves in situations of policy changes and being married, and then rate the following four statements on a five-point scale ranging from “never” to “always”: “How often would your job or career interfere with your family responsibilities (e.g., housework and childcare)?”, “How often would your job or career reduce the amount of time you would like to spend with your family?” “How often would your family life interfere with your responsibilities at work (e.g., getting to work on time, accomplishing daily tasks, or working overtime)?” and “How often would your family life reduce the amount of time you would like to spend on job or career-related activities?”. It should be noted that the intended scope of the work–family conflict questions is anticipatory rather than retrospective. This future projection is critical for understanding how individuals anticipate balancing work and family life, including the challenges they might expect to face following the arrival of a child. As three out of four questions are directly related to work or family time, the scale is a good fit for this research. Given the high internal consistency (Cronbach’s alpha = 0.85), the average score was calculated. The index of work–family conflicts ranges from 1 to 5 with a higher score indicating larger work–family conflicts.

Several pre-treatment characteristics are surveyed. First, we asked the respondents to report their occupations. Specifically, our measure of occupation is derived from the Singapore Standard Occupational Classification 2020^1^ and has the following major categories: Legislators, Senior Officials and Managers (1), Professionals (2), Associate Professionals and Technicians (3), Clerical Support Workers (4), Service and Sales Workers (5), Agricultural and Fishery Workers (6), Craftsmen and Related Trades Workers (7), Plant and Machine Operators and Assemblers (8), Cleaners, Labourers and Related Workers (9), Workers Not Elsewhere Classified, please specify (10). Second, considering that respondents may differ in the level of flexibility allowed for by their specific jobs, in addition to their current weekly numbers of regular work hours and overtime hours, we asked them to estimate the percentage of their jobs that is possible for flexible work schedule or workplace. Third, there are other covariates measuring the respondent’s demographic, socioeconomic, relationship, and job characteristics relevant to our study, such as the respondent’s age, gender, education, citizenship status, whether s/he currently has a partner. These covariates are important to determine if our randomization succeed in resulting in a balanced sample, in which the treatment and control groups do not differ on average statistically in these key (and hopefully, other related unmeasured) characteristics prior to treatment assignment.

It is worth mentioning that we are particularly interested in the occupational class as a theoretically sound moderator for understanding the heterogeneity of the FWAs’ effects. In our analysis, the first two occupational categories are combined as professional and managerial occupations (hereafter “professional occupation”), and the rest of the categories are combined as “non-professional occupations”. More specifically, the “Legislators, Senior Officials and Managers” category encompasses individuals in key governmental and corporate positions, such as parliament members, city council members, and top corporate executives like managing directors and CEOs. These roles involve creating laws and overseeing organizational strategies. The “Professionals” category includes highly trained individuals in specialized areas, including engineers, doctors, university professors, and business analysts. Both groups are characterized by their high socioeconomic status and deep expertise and significant contributions to their respective fields.

### Analytic strategy

Following previous research, we have used various analytic methods to examine fertility intentions. As fertility intention is an ordinal variable, we used the ordered logistic regression to maximize the use of available information and statistical efficiency. We first conduct full-sample analyses and then examine and compare similarities and differences in effect patterns specific to males and females, with separate sub-sample analyses in parallel. Next, to examine the mediation effect of work–family conflicts on FWAs’ treatment effects, we used the bootstrapping mediation method (Hayes, 2017). We obtained standard errors for testing the statistical significance of mediation effects using 200 bootstrap simulations. Finally, to examine the moderation effects of the respondent’s occupational class, we specify interaction terms between treatment conditions and occupational class. As both the treatment conditions and occupational class are categorical variables, statistically significant interaction effects between the two could be interpreted as systematic differences in treatment effects between occupational classes.

## Results

### Descriptive statistics and balance checks of the sample

Table 1 reports descriptive statistics about the above variables by experimental conditions. Overall, we find that in the control group while around 12% and 22% of respondents report definitely not or probably not having children in the next five years, 8% and 34% report definitely and probably having children. In contrast, those from the three treatment groups are much less likely to report low fertility intentions (definitely no: around 5–7%, probably no: around 12–17%) and more likely to report high fertility intentions (definitely yes: around 9–13%, probably yes: around 38–42%). The result is also consistent for the numerical measure of fertility intention. In the last column of Table 1, we report the p-values of the ANOVA F-test (to examine the association between the treatment variable and continuous variables) and Chi-squared test (to examine the association between the treatment variable and categorical variables). Overall, these test results show that both measures of fertility intentions significantly vary across the experimental conditions. Similarly, we observe that those from the treatment groups also have significantly higher marriage intentions than the control group, although the effect may be less pronounced for those in the “flexible schedule” group. Additionally, experiment groups tend to have lower average levels of anticipated work–family conflicts than the control group.

Meanwhile, we do not observe any statistically significant differences in answers to pre-treatment questions, indicating different treatment and control groups are balanced on pre-treatment covariates. These covariates include age, gender, partnership, citizenship, education levels, occupational status, number of weekly normal working hours, and number of weekly overtime hours. Namely, the randomization of our experiment has successfully eliminated all systematic differences between the control and treatment groups. In other words, the observed differences in the outcome variable, i.e., fertility intention, as well as the potential mediators, i.e., measures of anticipated work–family conflict, should amount to the treatment effects of future policy changes of FWAs. Additionally, we also followed previous research (Coibion et al., 2022, 2023) to examine whether respondents’ demographic and socioeconomic characteristics (e.g., age, gender, partnership, citizenship, education, and occupational characteristics) can predict the treatment variable by estimating a multinominal regression model in Table A2 in Appendix. Reassuringly, none of these variables is significantly related to our treatment variable, further confirming the success of our randomization.

**Table 2:** Ordered logistic regression models estimating the effects of flexible working arrangements on fertility intentions

	All	Men	Women
Treatment (Ref. = Control)			
Reduced hours	1.79***(0.29)	1.55 + (0.36)	2.05**(0.47)
Flexible schedule	1.55**(0.25)	1.42(0.33)	1.66*(0.37)
Flexible place	1.63**(0.28)	1.47(0.36)	1.79*(0.43)
Pseudo R-squared	0.02	0.01	0.02
Observations	1,092	537	555

Notes: Odds ratios are reported. Standard errors are in parentheses

^***^ p < 0.001, ** p < 0.01, * p < 0.05, + p < 0.1

### The treatment effects of the FWAs

Our experiment provides strong evidence that FWAs increase the respondent’s fertility intention. Table 2 reports our ordered logistic regression model of the effects of flexible working arrangements on fertility intention. Specifically, the odds of reporting high fertility intentions for those who received reduced hours, a flexible schedule, and a flexible workplace are 1.79 (p < 0.001), 1.55 (p < 0.01), and 1.63 (p < 0.01) times higher, respectively, compared to those in the control group. Moreover, the FWAs appear to have similar effect directions between men and women, but they may matter particularly to women as suggested by the relevant effect size and statistical significance. For men, we find that the arrangement of “reduced hours” can significantly improve men’s fertility intention, but only marginally significant at the 0.1 level. For women, we find that all three flexible working arrangements have statistically significant and positive effects on fertility intention (e.g., the odds ratio effect sizes range from 1.79 to 2.05, *p* < 0.05).

### The mediation of work–family conflicts

We examine whether anticipated work–family conflicts can mediate the effects of FWAs on fertility intention in Table 3. In panel A, for the pooled sample and the sub-sample of women, all three types of FWAs can significantly reduce anticipated work–family conflicts, whereas, for men, only reduced hours arrangement can reduce work–family conflicts. In panel B, bootstrapping mediation models show that in the pooled sample, work–family conflicts can significantly mediate the effects of all three FWAs. For men, work–family conflicts can only significantly mediate the effect of reduced hours arrangement (percent mediated = 31%). In contrast, for women, work–family conflicts can significantly mediate the effects of all reduced hours (percent mediated = 24%), flexible schedule (percent mediated = 33%), and flexible place arrangements (percent mediated = 39%).

**Table 3:** Bootstrapping mediation analyses of anticipated work–family conflicts

	All	Men	Women
Panel A. Anticipated work–family conflicts as dependent variable			
Treatment (Ref. = Control)			
Reduced hours	-0.31*** (0.07)	-0.33*** (0.09)	-0.27** (0.10)
Flexible schedule	-0.19** (0.07)	-0.13 (0.09)	-0.26** (0.09)
Flexible place	-0.23** (0.07)	-0.13 (0.10)	-0.33*** (0.10)
Constant	2.95*** (0.05)	2.90*** (0.07)	3.00*** (0.07)
R-squared	0.02	0.03	0.03
Observations	1,092	537	555
Panel B. Indirect Effect: Anticipated work–family conflicts as mediator of fertility intention			
Treatment (Ref. = Control)			
Reduced hours	0.23*** (0.05)	0.21** (0.07)	0.24** (0.07)
% mediated	35.24%	41.20%	31.21%
Flexible schedule	0.15** (0.05)	0.10 (0.06)	0.21** (0.07)
% mediated	29.94%	NA	38.76%
Flexible place	0.18*** (0.05)	0.11 (0.06)	0.24** (0.07)
% mediated	33.69%	NA	41.67%

Notes: Standard errors in parentheses calculated using 200 bootstrap simulations

^***^ p < 0.001, ** p < 0.01, * p < 0.05

### The moderation of occupational class

**Fig. 1:**
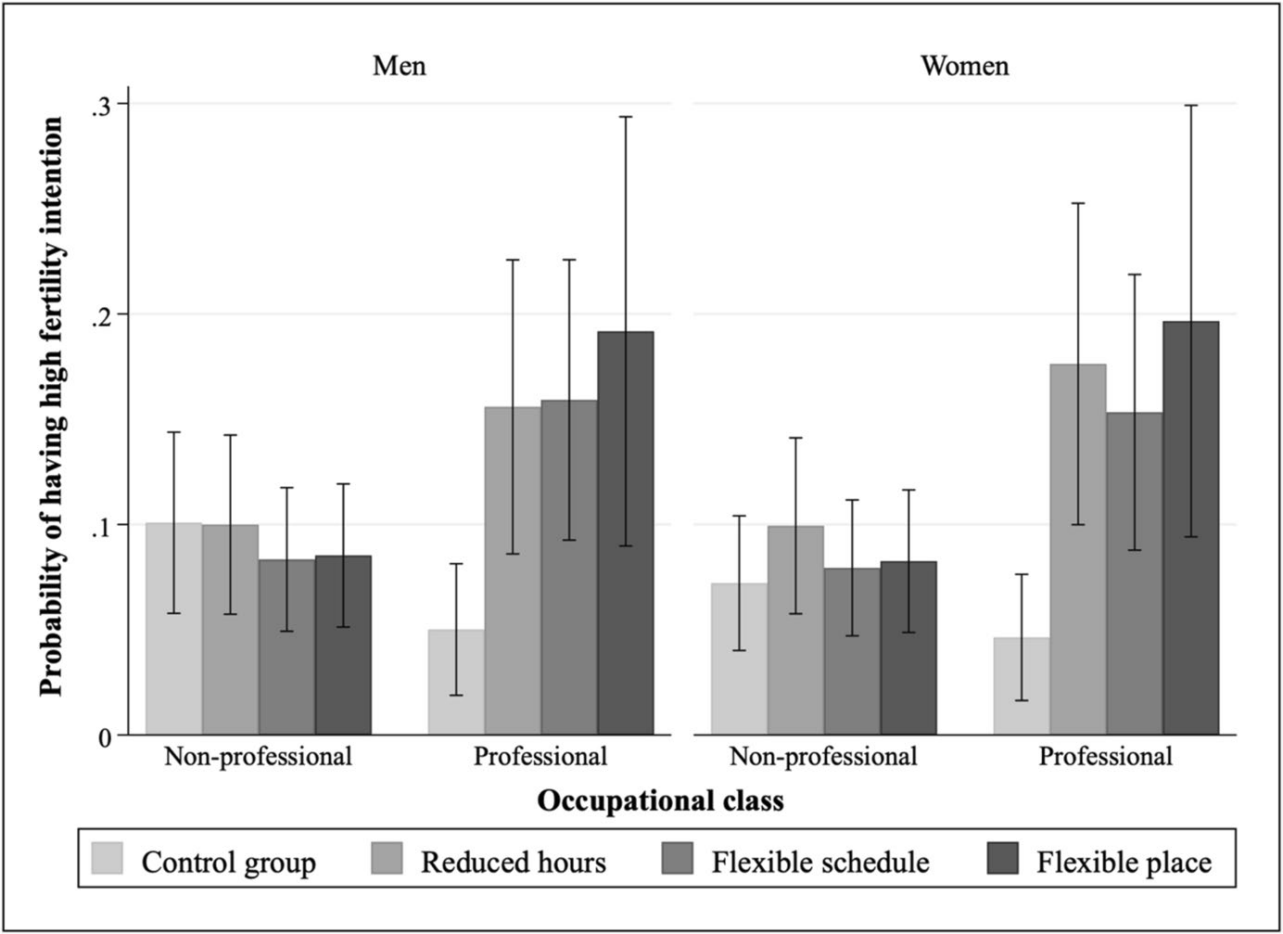
Predicted probability of having high fertility intention by status of flexible working arrangements and occupational class on with 95% confidence intervals

Figure 1 reports the interaction effects between FWAs and occupational class on fertility intention, with numerical results reported in Table A3. Overall, the interaction effects are statistically significant for both men and women, suggesting that the positive effects of FWAs on fertility intention are significantly more pronounced among those in professional occupations than in non-professional occupations. In non-professional occupations, respondents tend to have similar fertility intentions regardless of the flexible working treatment. In contrast, in the non-professional occupations, respondents in the control group tend to have particularly low fertility intention (even compared with those in non-professional occupations), but the disclosure of flexible working arrangements can significantly improve their fertility intention to a level, which is higher than fertility intentions of those in non-professional occupations.

### Supplementary analyses

Are the identified effects of FWAs reflect elevated marital intention rather than elevated fertility intention? In the presence of a culturally close correlation between marital and fertility intentions in the studied unmarried population, we conduct a supplementary analysis to ensure the validity of the results, which should not merely reflect the effects of FWAs on marital intention. In our survey experiment, we also measured marriage intention with the question: “Do you plan to get married within the next three years?” on the same scale as fertility intention. This question is asked after showing the treatment or control information. We repeat our main analysis while controlling the marriage intention, as reported in Table A4. The causal effects of FWAs on fertility intention remain statistically significant and of reasonably large magnitudes. Namely, while the FWAs may non-surprisingly influence an unmarried individual’s marriage intention at the same time, and the correlation between marriage and fertility intentions may be strong in our study population, FWAs have substantial effects on fertility intentions even after accounting for their potential influence on marriage intentions. In other words, although marital and fertility intentions are closely linked in our study population, what our experiment finds is not merely driven by the effects of FWAs on marriage intentions.

Besides, we have also examined other potential heterogeneities in the treatment effects of FWAs that are related to important aspects of the respondent’s current work arrangements. Recall that, in our survey, we asked about regular work hours, overwork hours, the percentage of work that potentially allows for flexible schedules, and the percentage of work that potentially allows for flexible workplaces of the respondent’s current job. As previously reported in our balance checks, thanks to successful randomization, the averages of these variables do not differ between treatment and control groups. However, do the treatment effects of FWAs vary according to these work arrangements? We repeat our moderation effect examinations and replace the moderator with these current work arrangements. As reported in Table A5, most of these current work arrangements are important moderators, suggesting the effects of FWAs may work similarly for individuals under different work arrangements. One exception is that for men the interactions between flexible schedules/workplaces and proportions of work allowing for flexible schedules/workplaces are positive and significant. This suggests that default policies of flexible schedules/workplaces are more likely to increase fertility intentions for men those current work allows more flexible schedules/workplaces.

Lastly, as the respondents were informed about the focus on “plans about future family lives”, this may lead to the experimenter demand effects, which refer to the changes in respondent responses due to cues about what constitutes appropriate behavior. This concern is always difficult to verify directly and comprehensively. To the best capability of our data, we conduct a partial robustness check to gauge this potential bias. We utilized a post-treatment question that measures the extent to which respondents are confident in government policy on a five-point scale ranging from “not confident at all” to “very confident”. We suppose that this variable could potentially help to reveal some possible experimenter demand effects, if any. The premise is that, given our treatment refers to scenarios of future policies, those who are more confident in government policy are probably more incentivized to manipulate their answers, hoping to be consistent with perceived governmental expectations or even influence future policymaking. Namely, we assume the respondent’s answer to this question partially correlates with potential experimenter demand effects. We have repeated our main models by controlling for respondents’ confidence in government policy. Overall, as reported in Table A6 the results remain similar to the main findings, suggesting a relatively low risk of experimenter demand effects.^2^

## Conclusion and Discussion

Despite a wide range of family policies, low fertility remains one of the most challenging social problems, which holds significant implications for long-term social cohesion and prosperity. While some scholars suggest that the prevalence of overwork norm may be a crucial reason for the low fertility rate calling for more legalized access to flexible working, there is little research on the effects of FWAs on fertility due to the endogenous relationship between work and family norms. Using a novel vignette survey experiment, this research aims to gain a better understanding of whether and how FWAs affect fertility intention in Singapore, a country with one of the lowest fertility rates and longest working hours in the world. Most importantly, we extend the attention to an important and under-studied population who are at reproductive ages and subject to FWAs policy changes directly—the unmarried population that have already accounted for over half of Singaporean population aged 20–39.

Overall, this study yields two important findings. First, we find that destabilizing the existing overwork norm through government-initiated FWAs policies can significantly increase young people’s fertility intention in Singapore. For women, this pattern is particularly pronounced with all three types of FWAs (i.e., reduced hours, flexible schedule, and flexible place) having significant effects on their fertility intention, whereas only reduced hours arrangement can significantly increase men’s fertility intention. The effects of different FWAs are comparable and about 30% can be mediated by lower anticipated work–family conflict. The findings are consistent with the literature that highlights overwork and work–family conflict as an institutional constraint preventing young people from developing intentions to have children, let alone achieving these intentions (Yeung & Hu, 2018). In contrast, legalized access to FWAs is found to help better accommodate conflicting responsibilities from work and family domains (especially for women) by enabling them to continue their career after childbirth and promoting a more equitable division of housework (Chung et al., 2021; Greenhaus & Beutell, 1985). As intention predicts behavior, we can expect that a more family-friendly working environment alongside other welfare policies could potentially improve the actual fertility rate in the long-term.

Nevertheless, there is still a large proportion of the effects unexplained by anticipated work–family conflict, which may be possibly attributed to other intervening mechanisms such as more leisure time and increased subjective wellbeing. In addition, for men, flexible schedule and flexible place may not have substantial effects and also cannot reduce their anticipated work–family conflict. This is consistent with the previous literature, which shows that while women primarily use FWAs for household-related purposes to maintain work–family balance, men tend to use flexible working primarily for performance-enhancing purposes and, therefore, work longer hours (Lott & Chung, 2016). This highlights the heterogeneous effects of different types of FWAs on work–family conflict across genders, which could have different implications for fertility intention.

Second, we find that the effects of FWAs on fertility intention are particularly pronounced among both men and women working in professional occupations. This is in line with our expectations and echoes the previous research that emphasizes the high fertility costs in the upper middle class (Brinton & Oh, 2019). On the one hand, strong overwork norm in the higher occupational class may lower fertility intention by increasing work–family conflict because it expects employees to work long hours and devote their time entirely to the job without interruptions from other non-work demands (Schieman et al., 2009a, 2009b). On the other hand, intensive parenting in the higher occupational class could not only exacerbate work–family conflict together with overwork norm, but also increase both material and opportunity costs of fertility (Lareau, 2011).

One possible reason that FWAs do not significantly improve fertility intentions among non-professionals could be related to their unique socioeconomic conditions (Kong & Dong, 2024;Yeung & Hu, 2018; Yeung & Yap, 2013). Non-professional workers may face more immediate and pressing concerns, such as income insecurity or financial instability, which may overshadow the benefits of FWAs. These workers are often in lower-paying jobs, where increasing income might take priority over work flexibility when considering family planning. Cultural factors could also play a role, as non-professional workers may adhere to more traditional family values, where fertility intentions are influenced by expectations of a conventional division of gender roles in the household, rather than workplace arrangements (Cha, 2010). Therefore, while FWAs may not be a top priority for this group now, they could become more relevant once their economic concerns are addressed. This highlights the need for further exploration of how different occupational groups prioritize various factors that influence fertility decisions.

Finally, this study has several limitations, calling for future research to follow up. First, this study only focused on fertility intention due to the nature of survey experiment design. Although fertility intention is highly related to fertility behavior, both are not equivalent. Also, the five-year span of fertility intention may be long for young people and this measure cannot distinguish between people intending to remain childless and those that are simply planning a childbirth later in life. Thus, future research could profitably incorporate various types of fertility desires, intentions and behavior into one framework to understand the whole family formation process.

Second, while FWAs are an important and under-studied determinant of fertility, there are other socioeconomic and cultural factors, which may jointly shape fertility intention. Future research could incorporate a wide range of factors in the experimental design to examine their relative and interactional effects. Third, despite our partial robustness check reported above, our study remains not entirely safe from potential experimenter demand effects. We suppose that in this study, such effects may not significantly bias our results given the rise of individualism and changing societal norms around family behavior. Specifically, as the decision to have children and the number of children are increasingly viewed as a matter of personal choice rather than adherence to social norms, the respondent responses in the study are less likely to be swayed by perceived expectations. This shift toward viewing fertility choices with less societal judgment may diminish the impact of experimenter demand effects, as respondents may feel less pressure to conform to any supposed experimental aims or societal expectations.

Lastly, as self-employed individuals already have some autonomy over their working conditions, the treatment of FWAs may not have the same relevance or impact for them as it does for standard employees.^3^ Unfortunately, we did not include a question about self-employment status in our questionnaire, which limits our ability to determine the proportion of self-employed individuals within each occupational category. We recognize this as a limitation and hope that future research can address this issue with a more refined design.

These limitations, however, cannot overshadow this study’s main contribution that destabilizing the existing overwork norm through government-initiated FWAs can enhance young people’s fertility intention, especially for those working in professional occupational class. Our study calls for a more family-friendly working environment to tackle low fertility rates in developed countries and holds important implications for understanding how family behavior may be planned in the future of work.

## Supplementary Information

Below is the link to the electronic supplementary material.

Supplementary file1 (DOCX 37 kb)

## Data Availability

The access to the data may need to be approved by the institution and/or the principal investigator. The data are classified as restricted data at the National University of Singapore (NUS Restricted). The definition of NUS Restricted is stated as the following: Documents, information, or materials other than those classified as NUS Confidential which are for use within NUS by authorized personnel on a need-to-know basis, including documents, information or materials which NUS has a contractual obligation to protect. Release of such documents, information, or materials may involve damage to the reputation and interests of NUS, or the individual or entity to which the documents, information or materials relate. For more information, see https://nusit.nus.edu.sg/its/resources/data-classification/#:~:text=The%20tool%20classifies%20office%20documents,recommended%20classification%20is%20deemed%20incorrect.

## Footnotes

1 For more information, see: https://www.singstat.gov.sg/standards/standards-and-classifications/ssoc

2 We thank one anonymous reviewer for pointing out this potential concern of “experimenter demand effects,” considering a specific wording in the questionnaire.

3 We would like to thank one anonymous reviewer for raising this important issue.
