# Supplementary material for: Flexible Working Arrangements and Fertility Intentions: A Survey Experiment in Singapore
Source: Eur J Popul. 2024 Nov 21;40(1):33. doi: 10.1007/s10680-024-09719-1 (PMC11582245; doi:10.1007/s10680-024-09719-1)
Supplement: Supplementary file 1 — Supplementary file1 (DOCX 37 kb) [file 10680_2024_9719_MOESM1_ESM.docx]

**Online Appendix**

| Table A1. Sample and census distributions in gender-age-education groups | | | |
| --- | --- | --- | --- |
| Gender-age-education groups | Unweighted sample % | Weighted sample % | Census % |
| Men aged 25-29 with low education levels | 11.54 | 13.41 | 13.41 |
| Men aged 25-29 with high education levels | 13.55 | 14.44 | 14.43 |
| Men aged 30-34 with low education levels | 5.04 | 7.07 | 7.08 |
| Men aged 30-34 with high education levels | 10.81 | 8.69 | 8.70 |
| Men aged 35-39 with low education levels | 2.84 | 4.46 | 4.46 |
| Men aged 35-39 with high education levels | 5.40 | 4.00 | 4.00 |
| Women aged 25-29 with low education levels | 13.74 | 8.13 | 8.13 |
| Women aged 25-29 with high education levels | 16.03 | 17.74 | 17.74 |
| Women aged 30-34 with low education levels | 4.76 | 4.34 | 4.33 |
| Women aged 30-34 with high education levels | 8.42 | 9.42 | 9.41 |
| Women aged 35-39 with low education levels | 2.11 | 3.20 | 3.21 |
| Women aged 35-39 with high education levels | 5.77 | 5.09 | 5.09 |
| Total (N = 1,092) | 100 | 100 | 100 |
| Note. % = Column proportions | | | |

| Table A2. Multinominal logistic regression models examining the associations between treatment and respondent socio-demographic characteristics. | | | |
| --- | --- | --- | --- |
| Reference group = Control | Reduced hours | Flexible schedule | Flexible place |
| Age groups (Ref. = 25-29) |  |  |  |
| 30-34 | 0.03 | -0.06 | 0.01 |
|  | (0.20) | (0.20) | (0.20) |
| 35-39 | -0.00 | 0.08 | 0.17 |
|  | (0.25) | (0.25) | (0.24) |
| Gender (Ref. = Male) | -0.13 | -0.06 | 0.04 |
|  | (0.17) | (0.17) | (0.17) |
| Partnership (Ref. = Yes) | -0.03 | 0.05 | -0.04 |
|  | (0.17) | (0.17) | (0.17) |
| Citizenship (Ref. = Citizens) | 0.39 | 0.08 | 0.32 |
|  | (0.25) | (0.26) | (0.25) |
| Education levels (Ref. = Below bachelor’s degree) | -0.08 | -0.14 | -0.13 |
|  | (0.19) | (0.18) | (0.18) |
| Occupation (Ref. = Non-professional) | 0.06 | 0.17 | -0.00 |
|  | (0.19) | (0.19) | (0.19) |
| Normal working time | -0.00 | -0.00 | 0.01 |
|  | (0.01) | (0.01) | (0.01) |
| Overtime | -0.01 | -0.00 | 0.01 |
|  | (0.02) | (0.02) | (0.02) |
| Proportion of work allowing flexible schedule | 0.00 | -0.00 | -0.00 |
|  | (0.00) | (0.00) | (0.00) |
| Proportion of work allowing flexible place | -0.00 | -0.00 | -0.00 |
|  | (0.00) | (0.00) | (0.00) |
| Observations | 1,092 | 1,092 | 1,092 |
| Note. Standard errors in parentheses. *** p<0.001, ** p<0.01, * p<0.05, + p<0.1 | | | |

| Table A3. Ordered logistic regression models examining the interaction effects between flexible working arrangements and occupational class on fertility intentions | | |
| --- | --- | --- |
|  | Men | Women |
| Panel A. Interaction between treatment and occupational class | | |
| Treatment (Ref. = Control) |  |  |
| Reduced hours | -0.01 (0.30) | 0.35 (0.27) |
| Flexible schedule | -0.21 (0.29) | 0.10 (0.27) |
| Flexible place | -0.18 (0.29) | 0.15 (0.28) |
| Occupational class (Ref. = Non-professional) | -0.75* (0.37) | -0.47 (0.37) |
| Treatment × Occupational class |  |  |
| Reduced hours × Professional | 1.26* (0.49) | 1.13* (0.49) |
| Flexible schedule × Professional | 1.49** (0.49) | 1.21* (0.49) |
| Flexible place × Professional | 1.69** (0.53) | 1.47** (0.53) |
| Pseudo R-squared  Observations | 0.04  537 | 0.05  555 |
| Note. Odds ratios are reported. Robust standard errors in parentheses. *** p<0.001, ** p<0.01, * p<0.05. | | |

| Table A4. Ordered logistic regression models examining the effects of flexible working arrangements and marriage intention on fertility intention | | | |
| --- | --- | --- | --- |
|  | All | Men | Women |
| Treatment (Ref. = Control) |  |  |  |
| Reduced hours | 1.59** (0.26) | 1.03 (0.25) | 2.44*** (0.54) |
| Flexible schedule | 1.68** (0.27) | 1.71* (0.42) | 1.63* (0.35) |
| Flexible place | 1.23 (0.21) | 1.18 (0.29) | 1.23 (0.30) |
| Marriage intention | 9.52*** (1.07) | 10.58*** (1.77) | 9.07*** (1.42) |
| Pseudo R-squared | 0.57 | 0.58 | 0.57 |
| Observations | 1,092 | 537 | 555 |
| Note. Odds ratios are reported. Robust standard errors in parentheses. *** p<0.001, ** p<0.01, * p<0.05, † p<0.1. | | | |

| Table A5. Ordered logistic regression models examining the interaction effects between flexible working arrangements, working time and work flexibility on fertility intentions | | | |
| --- | --- | --- | --- |
|  | All | Men | Women |
| Panel A. Interaction between treatment and working time | | | |
| Treatment (Ref. = Control) |  |  |  |
| Reduced hours | 2.70** (0.91) | 2.19 (1.06) | 3.13* (1.45) |
| Flexible schedule | 2.20** (0.66) | 2.37* (0.97) | 1.97 (0.83) |
| Flexible place | 1.96* (0.64) | 1.92 (0.90) | 2.06+ (0.88) |
| Overtime (Ref. = No) | 2.01** (0.55) | 1.71 (0.67) | 2.29* (0.83) |
| Treatment × Overtime |  |  |  |
| Reduced hours × Yes | 0.58 (0.22) | 0.63 (0.35) | 0.56 (0.29) |
| Flexible schedule × Yes | 0.63 (0.22) | 0.51 (0.25) | 0.80 (0.39) |
| Flexible place × Yes | 0.76 (0.29) | 0.69 (0.38) | 0.81 (0.41) |
| Pseudo R-squared | 0.03 | 0.02 | 0.04 |
| Observations | 1,092 | 537 | 555 |
| Panel B. Interaction between treatment and work schedule flexibility | | | |
| Treatment (Ref. = Control) |  |  |  |
| Reduced hours | 2.99* (1.28) | 4.04* (2.29) | 2.18 (1.45) |
| Flexible schedule | 2.13+ (0.85) | 3.79* (2.07) | 1.14 (0.70) |
| Flexible place | 1.69 (0.74) | 1.29 (0.81) | 2.12 (1.29) |
| Work schedule flexibility | 0.99* (0.00) | 0.98** (0.01) | 1.00 (0.01) |
| Treatment × Work schedule flexibility |  |  |  |
| Reduced hours × Work schedule flexibility | 1.01 (0.01) | 1.02+ (0.01) | 1.00 (0.01) |
| Flexible schedule × Work schedule flexibility | 1.00 (0.01) | 1.12* (0.01) | 0.99 (0.01) |
| Flexible place × Work schedule flexibility | 1.00 (0.01) | 1.00 (0.01) | 1.00 (0.01) |
| Pseudo R-squared | 0.03 | 0.05 | 0.03 |
| Observations | 1,092 | 537 | 555 |
| Panel C. Interaction between treatment and workplace flexibility | | | |
| Treatment (Ref. = Control) |  |  |  |
| Reduced hours | 1.91* (0.58) | 2.24+ (0.95) | 1.79 (0.82) |
| Flexible schedule | 1.89* (0.55) | 2.42* (0.97) | 1.61 (0.71) |
| Flexible place | 2.67** (0.83) | 2.97* (1.28) | 2.69* (1.26) |
| Workplace flexibility | 0.99 (0.00) | 0.98** (0.01) | 1.00 (0.01) |
| Treatment × Workplace flexibility |  |  |  |
| Reduced hours × Workplace flexibility | 1.00 (0.01) | 1.01 (0.01) | 1.00 (0.01) |
| Flexible schedule × Workplace flexibility | 1.00 (0.01) | 1.01 (0.01) | 1.00 (0.01) |
| Flexible place × Workplace flexibility | 1.10* (0.01) | 1.09* (0.01) | 1.01 (0.01) |
| R-squared | 0.02 | 0.04 | 0.03 |
| Observations | 1,092 | 537 | 555 |
| Note. Odds ratios are reported. Standard errors in parentheses. *** p<0.001, ** p<0.01, * p<0.05, + p<0.1. | | | |

| Table A6. Ordered logistic regression models examining the effects of flexible working arrangements on fertility intentions including the control of confidence in government policy | | | |
| --- | --- | --- | --- |
|  | All | Men | Women |
| Treatment (Ref. = Control) |  |  |  |
| Reduced hours | 1.65**(0.27) | 1.40 (0.32) | 1.97**(0.46) |
| Flexible schedule | 1.44*(0.23) | 1.22 (0.28) | 1.76**(0.38) |
| Flexible place | 1.56**(0.26) | 1.35 (0.32) | 1.85**(0.43) |
| Confidence in government policy | 1.83***(0.14) | 1.75***(0.19) | 1.93***(0.20) |
| Pseudo R-squared | 0.03 | 0.03 | 0.04 |
| Observations | 1,092 | 537 | 555 |
| Note. Odds ratios are reported. Standard errors in parentheses. *** p<0.001, ** p<0.01, * p<0.05, + p<0.1. | | | |

Survey Questionnaire

**Screening questions**

1. Are you a Singapore citizen or permanent resident?

◯ Singapore citizen (1)

◯ Singapore permanent resident (2)

◯ No (3) [Terminate]

◯ Prefer not to answer (4) [Terminate]

1. What is your current marital status?

◯ Never married

◯ Married [Terminate]

◯ Divorced [Terminate]

◯ Widowed [Terminate]

1. Do you have children?

◯ No

◯ Yes [Terminate]

◯ Prefer not to answer [Terminate]

1. Are you currently employed?

◯ No [Terminate]

◯ Yes

5. What is your age? [terminate if age<25 or age>39]

Answer:

(Range 25-39)

**Section 1. Demographic Background**

Q1. What is your sex?

◯ Male (1)

◯ Female (2)

◯ Prefer not to answer [Terminate]

Q2. What is your ethnicity?

◯ Chinese (1)

◯ Indian (2)

◯ Malay (3)

◯ Others (4)

◯ Prefer not to answer [Terminate]

Q3. Do you have a stable dating partner?

◯ Yes (1)

◯ No (2)

◯ Prefer not to answer [Terminate]

Q4. What is your highest education qualification obtained?

◯ No Formal Schooling (1)

◯ Primary (2)

◯ Secondary (3)

◯ Post-Secondary (Non-Tertiary): General and Vocational (4)

◯ Polytechnic Diploma (5)

◯ Professional Qualification and Other Diploma (6)

◯ Bachelor’s or Equivalent (7)

◯ Postgraduate Diploma/Certificate (Excluding Master’s and Doctorate) (8)

◯ Master’s and Doctorate or Equivalent (9)

Q5. What is your occupation?

◯ Legislators, Senior Officials and Managers (1)

◯ Professionals (2) (e.g., teacher, doctor, lawyer)

◯ Associate Professionals and Technicians (3) (e.g., accounting associate professionals. administrative and executive secretaries)

◯ Clerical Support Workers (4)

◯ Service and Sales Workers (5)

◯ Agricultural and Fishery Workers (6)

◯ Craftsmen and Related Trades Workers (7)

◯ Plant and Machine Operators and Assemblers (8)

◯ Cleaners, Laborers and Related Workers (9)

◯ Workers Not Elsewhere Classified, please specify.

Q6. What is your gross monthly income before tax deductions?

◯ Less than $ 3000 (1)

◯ $ 3000 – $ 4999 (2)

◯ $ 5000 – $ 6999 (3)

◯ $ 7000 – $ 8999 (4)

◯ $ 9000 – $ 10,999 (5)

◯ $ 11,000 or above (6)

◯ Prefer not to answer [Terminate]

Q7. What are your normal working hours per week excluding overtime?

Answer:

Q8. Beyond normal working hours, what are your overtime hours per week?

Answer:

Q9. How much of your regular work can be completed with a flexible schedule (e.g., flexible start and off work hours)?

◯ 0% ------------------------------------------------------- ◯ 100%

Q10. How much of your work can be completed outside of your regular workplace (e.g., work from home or preferred locations)?

◯ 0% ------------------------------------------------------- ◯ 100%

Q11. To what extent are you satisfied with your life?

◯ Completely dissatisfied (1)

◯ Mostly dissatisfied (2)

◯ Somewhat dissatisfied (3)

◯ Neither satisfied nor dissatisfied (4)

◯ Somewhat satisfied (5)

◯ Mostly satisfied (6)

◯ Completely satisfied (7)

Q12. Subjective status

Think of a ladder with 10 classes representing the socioeconomic position where people stand in Singapore. Please point the button to indicate your place on this status ladder.

◯ 1 (worst off class) ◯ 2 ◯ 3 ◯ 4 ◯ 5 ◯ 6 ◯ 7 ◯ 8 ◯ 9 ◯ 10 (best off class)

Q13. What is your assessment about how income (the total sum of annual earnings) is distributed in Singapore?

◯ Extremely unequally distributed (1)

◯ Very unequally distributed (2)

◯ Rather unequally distributed (3)

◯ Rather equally distributed (4)

◯ Very equally distributed (5)

Q14. To what extent do you agree with the following statements?

A. Husbands should be more responsible for financially supporting the family than wives.

◯ 1 (strongly agree) ◯ 2 ◯ 3 ◯ 4 ◯ 5 ◯ 6 ◯ 7 (strongly disagree)

B. Wives should be more responsible for housework and/or childcare than husbands.

◯ 1 (strongly agree) ◯ 2 ◯ 3 ◯ 4 ◯ 5 ◯ 6 ◯ 7 (strongly disagree)

**Section 2. Experiment**

The next section will show you some government regulations of working time and possible future changes. Please read the text and table in the next page carefully!

**Vignette 1**

**Please read the following text and table carefully.**

In Singapore, the average working hours are 44.6 hours per week with 23 percent of people working more than 48 hours per week. Usually, the default work arrangement is working from 9am to 6pm for 5 days per week at the designated workplace. Currently, the government has no national-level policies entitling employees legal rights to adjust their working schedule or place flexibly. Given the existing policies (see the Table below), we are interested in learning about you plans about future family lives.

| **Labor law regulations** |
| --- |
| 1. Standard working hours |
| Current regulations: 44 hours per week |
| 1. Flexible work schedule |
| Current regulations: No legal regulations |
| 1. Flexible workplace |
| Current regulations: No legal regulations |

**Vignette 2**

**Please read the following text and table carefully.**

In Singapore, the average working hours are 44.6 hours per week with 23 percent of people working more than 48 hours per week. Usually, the default work arrangement is working from 9am to 6pm for 5 days per week at the designated workplace. Currently, the government has no national-level policies entitling employees legal rights to adjust their working schedule or place flexibly. In the foreseeable future, if the government would **reduce the standard working hours per week from 44 to 36 hours,** **while keeping the income level and other work arrangements unchanged** (see the Table below), we are interested in learning about your plans about future family lives.

| **Labor law regulations** |
| --- |
| 1. Standard working hours |
| Current regulations: 44 hours per week |
| **Future changes: 36 hours per week** |
| 1. Flexible work schedule |
| Current regulations: No legal regulations |
| Future changes: No change |
| 1. Flexible workplace |
| Current regulations: No legal regulations |
| Future changes: No change |

**Vignette 3**

**Please read the following text and table carefully.**

In Singapore, the average working hours are 44.6 hours per week with 23 percent of people working more than 48 hours per week. Usually, the default work arrangement is working from 9am to 6pm for 5 days per week at the designated workplace. Currently, the government has no national-level policies entitling employees legal rights to adjust their work schedule or place flexibly. In the foreseeable future, if the government would **entitle employees to flexibly adjust their work schedule (e.g. start and end working times) for at least two working days per week, while keeping the income level and other work arrangements unchanged** (see the Table below), we are interested in learning about your plans about future family lives.

| **Labor law regulations** |
| --- |
| 1. Standard working hours |
| Current regulations: 44 hours per week |
| Future changes: No change |
| 1. Flexible work schedule |
| Current regulations: No legal regulations |
| **Future changes: Flexible work schedule for at least two days per week** |
| 1. Flexible workplace |
| Current regulations: No legal regulations |
| Future changes: No change |

**Vignette 4**

**Please read the following text and table carefully.**

In Singapore, the average working hours are 44.6 hours per week with 23 percent of people working more than 48 hours per week. For most companies during the normal time, the default working mode is working from 9am to 6pm for 5 days at the company office. Currently, the government does not have any national-level policies giving employees legal rights to adjust their working schedule or place flexibly. In the foreseeable future, if the government would **entitle eligible employees to flexibly adjust their work place (e.g., working from home / preferred locations) for at least two days per week, while keeping the income level and other work arrangements unchanged** (see the Table below), we are interested in learning about your plans about future family lives.

| **Labor law regulations** |
| --- |
| 1. Standard working hours |
| Current regulations: 44 hours per week |
| Future changes: No change |
| 1. Flexible work schedule |
| Current regulations: No legal regulations |
| Future changes: No change |
| 1. Flexible workplace |
| Current regulations: No legal regulations |
| **Future changes: Flexible workplace for at least two days per week** |

Q15. Given the aforementioned policy scenario of **[insert text of experimental condition respondent was assigned to]**, do you plan to get married within the next three years?

◯ Definitely yes (1)

◯ Probably yes (2)

◯ Not sure (3)

◯ Probably no (4)

◯ Definitely no (5)

Q16. Given the aforementioned policy scenario of **[insert text of experimental condition respondent was assigned to]**, do you plan to have a child within the next five years?

◯ Definitely yes (1)

◯ Probably yes (2)

◯ Not sure (3)

◯ Probably no (4)

◯ Definitely no (5)

Q17. Given the aforementioned policy scenario of **[insert text of experimental condition respondent was assigned to]**, were you in marriage, how often would your job or career interfere with your family responsibilities (e.g. housework and childcare)?

◯ Never (1)

◯ Seldom (2)

◯ Sometimes (3)

◯ Usually (4)

◯ Always (5)

Q18. Given the aforementioned policy scenario of **[insert text of experimental condition respondent was assigned to]**, were you in marriage, how often would your job or career reduce the amount of time you would like to spend with your family?

◯ Never (1)

◯ Seldom (2)

◯ Sometimes (3)

◯ Usually (4)

◯ Always (5)

Q19. Given the aforementioned policy scenario of **[insert text of experimental condition respondent was assigned to]**, were you in marriage, how often would your family life interfere with your responsibilities at work (e.g. getting to work on time, accomplishing daily tasks, or working overtime)?

◯ Never (1)

◯ Seldom (2)

◯ Sometimes (3)

◯ Usually (4)

◯ Always (5)

Q20. Given the aforementioned policy scenario of **[insert text of experimental condition respondent was assigned to]**, were you in marriage, how often would your family life reduce the amount time you would like to spend on job or career-related activities?

◯ Never (1)

◯ Seldom (2)

◯ Sometimes (3)

◯ Usually (4)

◯ Always (5)

**Section 3. Robustness and manipulation checks**

Q21. Please recall, which of the following policy scenario have you seen in the previous section?

◯ Existing working arrangements (1)

◯ Reducing standard working hours (2)

◯ Entitling flexible work schedule (3)

◯ Entitling flexible workplace (4)

Q22. To what extent are you confident in Singapore government policy?

◯ Not confident at all (1)

◯ Of little confidence (2)

◯ Of average confidence (3)

◯ Very confident (4)

◯ Absolutely confident (5)
